# A modified TILLING approach to detect induced mutations in tetraploid and hexaploid wheat

**DOI:** 10.1186/1471-2229-9-115

**Published:** 2009-08-28

**Authors:** Cristobal Uauy, Francine Paraiso, Pasqualina Colasuonno, Robert K Tran, Helen Tsai, Steve Berardi, Luca Comai, Jorge Dubcovsky

**Affiliations:** 1 Department of Plant Sciences, University of California, Davis, CA, 95616, USA; 2 Department of Genetics and Plant Breeding, University of Bari, Italy; 3 UC Davis Genome Center, University of California, Davis, CA, 95616, USA; 4 John Innes Centre, Colney, Norwich NR4 7UH, UK

## Abstract

**Background:**

Wheat (*Triticum *ssp.) is an important food source for humans in many regions around the world. However, the ability to understand and modify gene function for crop improvement is hindered by the lack of available genomic resources. TILLING is a powerful reverse genetics approach that combines chemical mutagenesis with a high-throughput screen for mutations. Wheat is specially well-suited for TILLING due to the high mutation densities tolerated by polyploids, which allow for very efficient screens. Despite this, few TILLING populations are currently available. In addition, current TILLING screening protocols require high-throughput genotyping platforms, limiting their use.

**Results:**

We developed mutant populations of pasta and common wheat and organized them for TILLING. To simplify and decrease costs, we developed a non-denaturing polyacrylamide gel set-up that uses ethidium bromide to detect fragments generated by crude celery juice extract digestion of heteroduplexes. This detection method had similar sensitivity as traditional LI-COR screens, suggesting that it represents a valid alternative. We developed genome-specific primers to circumvent the presence of multiple homoeologous copies of our target genes. Each mutant library was characterized by TILLING multiple genes, revealing high mutation densities in both the hexaploid (~1/38 kb) and tetraploid (~1/51 kb) populations for 50% GC targets. These mutation frequencies predict that screening 1,536 lines for an effective target region of 1.3 kb with 50% GC content will result in ~52 hexaploid and ~39 tetraploid mutant alleles. This implies a high probability of obtaining knock-out alleles (*P *= 0.91 for hexaploid, *P *= 0.84 for tetraploid), in addition to multiple missense mutations. In total, we identified over 275 novel alleles in eleven targeted gene/genome combinations in hexaploid and tetraploid wheat and have validated the presence of a subset of them in our seed stock.

**Conclusion:**

We have generated reverse genetics TILLING resources for pasta and bread wheat and achieved a high mutation density in both populations. We also developed a modified screening method that will lower barriers to adopt this promising technology. We hope that the use of this reverse genetics resource will enable more researchers to pursue wheat functional genomics and provide novel allelic diversity for wheat improvement.

## Background

Wheat is an important food crop that is grown worldwide and provides approximately 20% of the calories consumed by mankind [1]. In spite of its economic importance, the ability to modify and understand gene function in wheat is still not fully developed due to several limitations. The large size of the wheat genome (16,000 Mb in hexaploid wheat) [2] and its high content of repetitive DNA (83%) [3] are important obstacles for the complete genome sequencing of wheat. In addition, wheat is a polyploid species with most genes represented by two (in tetraploid) or three (in hexaploid) homoeologous copies that share approximately 93–96% sequence identity. Gene duplication limits the use of forward genetics phenotypic screens as the effect of single-gene knockouts are frequently masked by the functional redundancy of homoeologous genes present in the other wheat genomes [4].

Despite these barriers, a broad range of genomic resources have been developed for wheat. Over one million expressed sequence tags (EST) are deposited in GenBank covering ~60% of the expressed genome [5]. Multiple diploid, tetraploid, and hexaploid bacterial artificial chromosome (BAC) libraries [6-10] have been constructed in wheat and colinearity has been established between wheat and the sequenced rice [11] and *Brachypodium *genomes [12]. These resources have facilitated the positional cloning of several agronomically important genes, but the functional validation of the candidate genes has relied mainly in transgenic approaches that are laborious, low throughput and require regulatory oversight. The recent assembly of the chromosome 3B physical map [13] provides a feasible strategy for a chromosome-based approach for the future sequencing of wheat. The anchored contigs of chromosome 3B will provide the initial template for the sequencing of this chromosome generating an unprecedented amount of sequence information in wheat.

The ability to determine the function of these and other genes will ultimately depend on the establishment of robust, flexible and high-throughput reverse genetic tools. Reverse genetic approaches use sequence information to identify candidate genes and then study the phenotype of the mutant alleles to determine gene function. Several techniques are currently used for this purpose. T-DNA or transposon insertional mutagenesis has been used successfully in rice and Arabidopsis to assemble large gene knockout collections [14-16], but has not been extended to wheat. RNA interference is also a valuable technique in wheat since multiple homoeologues can be simultaneously down-regulated (reviewed in [17]), but it is a time-consuming procedure that must be designed specifically for the genes of interest. In addition, both techniques are based on transgenic transformation which is limited to few varieties in wheat, is subject to strict regulatory controls, and is not currently being used for crop improvement.

Recently, a powerful reverse genetics approach was implemented in wheat through the combination of ethyl methane sulphonate (EMS)-mediated mutagenesis and TILLING technology [18]. Briefly, a TILLING screen starts with PCR amplification of a target region from pooled DNA of mutagenized plants. This is followed by a mismatch-specific endonuclease digestion that is visualized by size-separation on polyacrylamide or agarose gels to identify mutant individuals. Once a positive individual is found it is sequenced to determine the exact mutation it carries. Gene function is assigned based on phenotypic evaluation of the mutant individuals.

TILLING is a flexible reverse genetics approach that generates a lasting resource that can be utilized to screen multiple targets. EMS-mediated mutagenesis is efficient in different genetic backgrounds allowing cultivar-specific libraries to be constructed according to the required needs. Alleles generated by TILLING can be readily used in traditional breeding programs since the technology is non-transgenic and the mutations are stably inherited. These advantages are reflected by the successful implementation of TILLING in several plant species such as Arabidopsis [19], maize [20], wheat [18,21], barley [22], rice [23,24], pea [25], potato [26], *Lotus japonicus *[27] and soybean [28].

Most TILLING systems rely on the use of high-throughput genotyping platforms, such as LI-COR gene analyzers, which use fluorescently labeled primers and are relatively expensive setups for individual laboratories. The investment and technical skills required for TILLING could be barriers to the adoption of this technology. Recently, agarose based detection systems have been suggested as inexpensive alternatives to the current technology intensive platforms [29,30], and its use for detecting EMS-induced mutations in large libraries has recently been determined [21].

The ability to understand gene function will become increasingly important as more sequence information is generated in wheat. Thus, there is a need for a diverse set of publicly available reverse genetic resources in wheat to assist with the functional validation of candidate genes. We report here the construction of two TILLING libraries from tetraploid and hexaploid wheat and their characterization through the TILLING of multiple targets. We developed a modified detection method based on polyacrylamide gel staining with ethidium bromide to make this technology more accessible and describe strategies for TILLING in polyploid genomes.

## Results

### Generation of EMS mutagenized population

We developed TILLING populations in tetraploid and hexaploid wheat using EMS as a chemical mutagen. For the tetraploid population we mutagenized seeds of the Desert durum^® ^variety 'Kronos', which was developed by Arizona Plant Breeders from a male sterile population (selection D03–21). For the hexaploid TILLING population we used the Hard Red Spring common wheat breeding line 'UC1041+*Gpc-B1*/*Yr36*'. UC1041 is a short stature breeding line developed by the University of California from the cross Tadinia/Yecora Rojo. 'UC1041+*Gpc-B1*/*Yr36*' was developed later by backcrossing for six generations a 6BS chromosome segment from *T. turgidum *ssp. *dicoccoides *that carries the high grain protein gene *Gpc-B1 *[31] and the partial stripe rust resistance gene *Yr36 *[32]. The EMS concentrations used to mutagenize the populations were 0.7 to 0.75% (57 to 60 mM) for Kronos and 0.9 to 1.0% (73 to 80 mM) for 'UC1041+*Gpc-B1*/*Yr36*'. Similar EMS concentrations have been used previously to create TILLING population in wheat [18,21]. Germination rates for EMS-treated seeds were ~50–60% (results not shown). We extracted DNA from single M_2 _plants and collected their M_3 _seeds to have independent and non-redundant mutations in our libraries. DNAs from a total of 1,368 M_2 _(tetraploid) and 1,536 M_2 _(hexaploid) plants were pooled in groups of four DNAs and organized into four 96-well plates for convenient screening (342 and 384 4× pools in the tetraploid and hexaploid populations, respectively). The tetraploid TILLING population is currently being expanded to 1,536 lines.

### Development of genome specific primers

We characterized the TILLING libraries by screening for mutations in the two *Starch Branching Enzyme II *genes, *SBEIIa *and *SBEIIb*, for tetraploid wheat and for mutations in the *Wheat Kinase Start *(*WKS*) *1*, *WKS2 *and *SBEIIa *genes in hexaploid wheat. *WKS1 *and *WKS2 *are single copy genes on chromosome arm 6BS [32], so there was no need to develop genome specific primers. *SBEIIa *and *SBEIIb *map to chromosome 2 and have homoeologous loci in each of the different wheat genomes. To screen for mutations in each of the homoeologous copies we designed primers specific for each copy taking advantage of polymorphic indels and SNPs between the different homoeologues. We designed primers complementary to intron sequences flanking the target exons and positioned approximately ~200-bp from the sequence of interest. The genome specificity of the *SBEII *primers was validated using nulli-tetrasomic lines of chromosome 2 (N2AT2B, N2BT2D, N2DT2A), DNAs from BAC clones from the A and B genomes obtained from a tetraploid BAC library [8] and *Aegilops tauschii *genomic DNA.

### Wheat TILLING platform using the Non-denaturing Polyacrylamide Detection Method

The screening of a TILLING population includes three fundamental steps: an initial screen of DNA pools to identify those that contain mutant individuals; a second screen to identify the individual within each pool that contains a putative mutation; and lastly, confirmation of the individual mutations by sequencing of the PCR products. Although some modifications exist, such as directly sequencing all individuals from a positive pool, most TILLING approaches follow this general framework.

**Figure 1:**
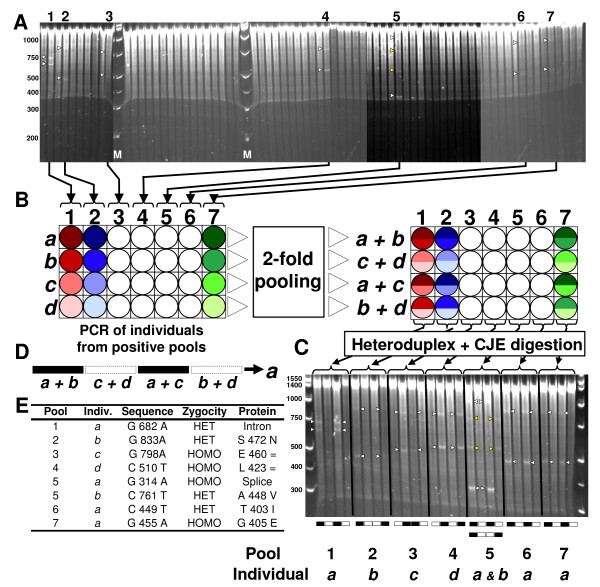
**TILLING using a non-denaturing polyacrylamide detection method**: **A) **Visualization of four-fold DNA pools digested with CJE after running on a non-denaturing 3% polyacrylamide gel for 75 minutes. Putative mutations in the pools are identified by the presence of two bands (indicated by white arrows) whose sizes add up to the full length PCR product. In pool 5, more than two bands are visible, representing two mutations within this pool (yellow arrows). Size markers (M) are included throughout the gel. This is a composite of four images whose contrast has been adjusted differently to allow better visualization. **B) **For each positive pool (labeled 1 through 7), the four individual DNAs (labeled *a *through *d*) are organized in a 96-well plate and used for PCR amplification of the target region. After PCR, paired pools are assembled by combining 6 μl of PCR product from two individuals and organizing them into a new 96-well plate. For example, row *a+b *contains 6 μl from individual *a *and 6 μl from individual *b*. **C) **Heteroduplexes are formed through denaturing and annealing of the pooled PCR products and mismatches were digested with CJE. Cleaved fragments were visualized using the non-denaturing polyacrylamide gel electrophoresis set-up as before. Each column is run in adjacent lanes, such that the first four lanes contain the four two-fold pools (*a+b*, *c+d*, *a+c *and *b+d*) from column 1. True mutations are replicated in two separate gel lanes within each set of four, producing a unique banding pattern (represented below each set of four lanes and represented in panel **D**). According to this pattern, the mutation can be unequivocally assigned to one of the individual DNAs. **E) **The PCR product from these individuals (leftover from the PCR on panel **B**) is sequenced and the identity of the mutation is determined.

We developed a TILLING platform that uses a non-denaturing polyacrylamide detection method to perform the two rounds of screening. After digestion of mismatches in heteroduplexes with celery juice extract (CJE), the fragments resulting from dsDNA cuts at mismatched sites are separated in native polyacrylamide gels and visualized through the fluorescence of bound ethidium bromide. For the initial step, the screen identifies the 4× pools with mutant individuals (Figure 1A). Targets are amplified by PCR from the four genomic DNAs that comprise each positive pool (*a, b, c, d *in Figure 1B). PCR products are then combined in four two-fold pools, heated and annealed to achieve heteroduplex formation, and finally digested with CJE (Figure 1C). Depending on the banding pattern (Figure 1C), the mutation is assigned to one of the four individual DNAs (Figure 1D, only one banding pattern example is shown). The mutant individual is then sequenced and the identity of the mutation is established (Figure 1E). A more detailed description can be found in the Methods section.

This method provides an independent validation of the mutation, identifies its location within the target region, and determines which individual from the pool carries the mutation. The paired pooling (Figure 1B) is necessary to detect homozygous mutations in the M_2 _plants since combining two samples allows heteroduplex formation and detection. This strategy also reduces the number of false positive errors as a true mutation should be observed in two separate gel lanes (Figure 1C).

Screening of *WKS1*, *WKS2 *and *SBEIIa *in the hexaploid library yielded 71, 50 and 65 mutations, respectively (Table 1). This translates into an estimated mutation density of at least one mutation per 49.4 kb screened in the hexaploid library. In the tetraploid library, we detected 58 and 35 mutants for *SBEIIa *and *SBEIIb*, respectively. Using a similar analysis as above, the estimated mutation density in the tetraploid library is at least one mutation per 68 kb screened. The relevance of these mutations was recently confirmed by Fu *et al*. [32] who used the *WKS1 *missense and *WKS2 *nonsense mutations (Table 2) to validate a candidate gene for broad-spectrum disease resistance. For the *SBE *genes (Table 3) we have identified and selected mutants that include splice junction mutations, a premature stop codon and several missense mutations that are predicted to have an effect on SBE protein activity. We have initiated the backcrossing of the mutants into non-mutagenized lines of Kronos and UC1041 to reduce the mutation load of the lines for future phenotypic analysis. The ability to identify truncation mutations in five of the seven *SBE *targets and putative non-functional amino acid substitutions in the remaining genes highlights the power of this approach for functional gene analysis.

**Table 1:** Characteristics of TILLING targets and mutation frequencies in the hexaploid and tetraploid TILLING populations

**Gene**	**Pop**.	**Chr**.	**Size (bp)**	**GC content (%)**	**M**_2_**plants screened**	**Mutations**	**Mutation Frequency**
*WKS1*	6×	6B	1371	39.8	1536	28	1/60 kb
	6×	6B	1270	40.7	1536	43	1/37 kb^a^
*WKS2*	6×	6B	1460	39.1	768	25	1/36 kb
	6×	6B	1532	39.8	768	25	1/42 kb^b^
*SBEIIa*	6×	2A	1593	37.7	1536	40	1/49 kb
	6×	2B	1638	37.7	768	17	1/59 kb
	6×	2D	1614	37.2	768	8	1/124 kb

*SBEIIa*	4×	2A	1637	37.9	1368	31	1/58 kb
	4×	2B	1641	37.8	1368	27	1/67 kb
*SBEIIb*	4×	2A	1909	36.8	600	15	1/61 kb
	4×	2B	1972	36.8	1152	20	1/91 kb

^a ^384 M_2 _plants were screened with LI-COR and 1152 M_2 _plants were screened using the polyacrylamide/ethidium bromide method.

^b ^All 768 M_2 _plants were screened with LI-COR.

**Table 2:** PSSM and SIFT scores of *WKS *mutations.

**Gene**	**Domain**	**Line**	**Nucleotide Change**	**Amino Acid Change**	**PSSM**	**SIFT**	**Reaction to PST**
*WKS1*	Kinase	T6-569	G 163 A	V 55 I	11.5	0.00	Susceptible
		T6-89	G 508 A	D 170 N	10.4	0.46	Resistant
		T6-312	G 585 A	G 199 R	19.7	0.00	Susceptible
		T6-480-1	C 632 T	T 211 I	12.6	0.01	Susceptible
		T6-138	G 914 A	R 305 H	13.6	0.01	Susceptible
	START	T6-567	G 4437 A	D 477 N	12.3	0.00	Susceptible
*WKS2*	Kinase	T6-960	C 13 T	R 5 *	---^a^	---	Resistant
		T6-480-2	G 72 A	W 24 *	---	---	Resistant
	START	T6-826	G 2221 A	W 379 *	---	---	Resistant

^a ^PSSM and SIFT scores are not reported for mutations that cause premature stop codons

Six *WKS1 *and three *WKS2 *mutants were scored as susceptible or resistant based on their reaction to *Puccinia striiformis *f. sp. *tritici *(PST) in Fu *et al*. [32]. In the nucleotide/amino acid change columns, the first letter indicates the wild type nucleotide/amino acid, the number its position from the start codon/methionine, and the last letter the mutant nucleotide/amino acid. High PSSM (>10) and low SIFT scores (<0.05) predict mutations with severe effects on protein function.

**Table 3:** Summary of selected *SBE *mutations.

**Gene**	**Pop**.	**Genome**	**Line**	**Nucleotide Change**	**Amino Acid Change**	**PSSM**	**SIFT**
*SBEIIa*	6×	A	T6-360	G 799 A	E 232 K	16.1	0.00
		A	T6-726	G 385 A	G 211 S	18.5	0.00
		A	T6-110	C 964 T	S 259 F	19.4	0.00
		B	T6-111	G 860 A	Splice Junction	---^a^	---
		D	T6-630	G 850 A	Splice Junction	---	---
	4×	A	T4-2179	G 401 A	W 216 *	---	---
		B	T4-1214	G 1347 A	Splice Junction	---	---
*SBEIIb*	4×	A	T4-385	G 1281 A	Splice Junction	---	---
		A	T4-1344	G 1121 A	Splice Junction	---	---
		A	T4-2574	G 308 A	Splice Junction	---	---
		B	T4-508	C 1290 T	P 283 L	19.5	0.01

^a ^PSSM and SIFT scores are not reported for mutations that cause premature stop codons or splice junction mutations

In the nucleotide change column, the position is relative to the forward primer used for the specific target since we do not have the complete genomic sequence for all *SBE *genes. In the amino acid change column, the position is relative to the start methionine based on the predicted amino acid sequence of the *Ae. tauschii *sequence [SBEIIa: GenBank AF338431, SBEIIb: GenBank AY740398].

In an M_2 _population, 33% of the mutations are expected to be found in homozygous state. For both populations we had a slight bias towards homozygous mutations, 37% in hexaploid and 42% in tetraploid, although these percentages were not significantly different from the expected 33% (hexaploid χ^2 ^= 1.18, *P *= 0.28; tetraploid χ^2 ^= 3.09, *P *= 0.08). Sequencing also confirmed that over 99% of the mutations were G to A or C to T transitions as expected from alkylation by EMS, with only one exception in the *SBEIIa *A genome target which was a C to G transversion (T6-2312).

**Figure 2:**
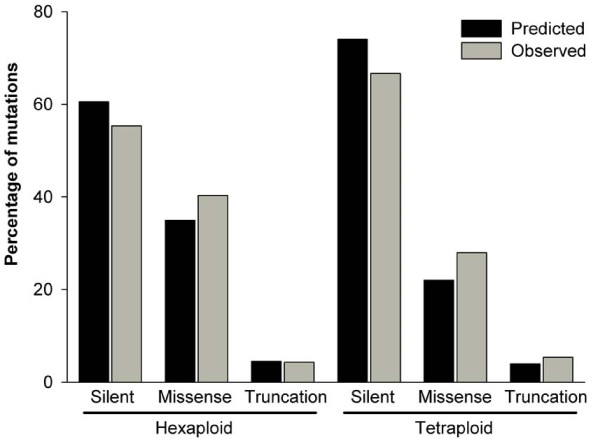
**Comparison of predicted and observed mutation types in the TILLING populations**. All mutation types were classified as either silent (synonymous mutations or within introns), missense (non-synonymous amino acid change) or truncation (splice junction mutations or nonsense). The predicted effects for each amplicon were calculated using CODDLE and considers all possible EMS mutations within the target region. The observed percentages describe the effects of all mutations in the hexaploid (*N *= 186 mutations) and tetraploid (*N *= 93 mutation) populations.

Using the CODDLe (Choose codons to Optimize the Detection of Deleterious Lesions) program [33], we predicted the effect of EMS mutations in the different amplicons. In the hexaploid library we identified a total of 186 mutations of which 40% were missense and 4.3% were truncations (nonsense or splice junction mutations). The predicted effects by CODDLE were 35% missense and 4.5% truncations, very close to the observed values (Figure 2). In the tetraploid screen we identified 93 mutations of which 28% were missense and 5.4% were truncations, whereas CODDLE predicted 22% missense mutations and 3.9% truncations. For both libraries, the distribution of silent, missense and truncation mutations were not significantly different from those predicted by CODDLE (tetraploid χ^2 ^= 2.37, *P *= 0.31; hexaploid χ^2 ^= 2.66, *P *= 0.26).

Several of the mutations in the *WKS *and *SBE *genes were identified in more than one independent individual. In the hexaploid library, 11.8% of the mutations were found in duplicate or triplicate (8 duplicate and 2 triplicate mutations, or 22 mutations in 186), which was higher (*P *< 0.01) than the expected 6.5% calculated using a Poisson distribution and the number of potential GC sites in the screened region. In the tetraploid library 24.7% of the mutations were found in more than one individual (8 duplicate, 1 triplicate, 1 quadruple, or 23 mutations in 93). These numbers were again higher (*P *< 0.01) than the expected 3.4% predicted by a Poisson distribution.

### Comparison between LI-COR and Non-denaturing Polyacrylamide Detection Method

Laser detection of fluorescently labeled DNA fragments, using a LI-COR genotyping platform, is the most widely used detection method to screen TILLING populations for mutations. To evaluate an alternative to this detection method, we screened two regions in both *WKS1 *and *WKS2 *using a non-denaturing 3% polyacrylamide set-up (Figure 1) and compared the results with an established LI-COR platform.

**Figure 3:**
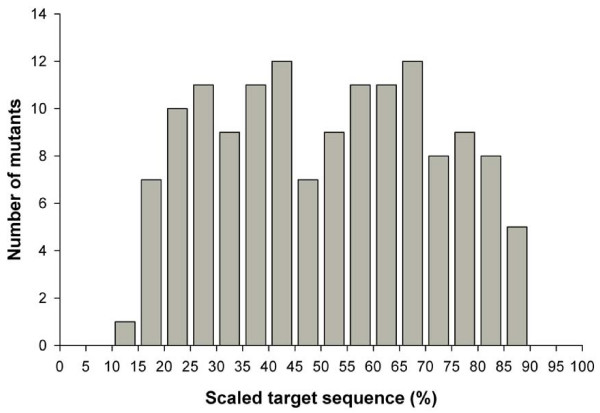
**Distribution of mutations detected by the polyacrylamide/ethidium bromide platform within the target sequence**. The position of each confirmed mutation (*N *= 141) in the seven targeted gene/genome combinations in hexaploid wheat is plotted against the target sequence scaled to 100%, with each bin representing 5% of the target sequence. No mutations were detected in the first and last two bins (0–10% and 90–100%) which represent the sequence closest to either forward or reverse primers.

TILLING of the same four *WKS *targets in 768 M_2 _individuals from the hexaploid library revealed that these two methods detect comparable number of mutations (Table 4). We used the method of Greene *et al*. [34] to estimate mutation densities (cumulative length of sequence screened divided by total number of mutants). We adjusted the total length of each target as mutations in the regions closest to the primers are not readily detected by either method. For the LI-COR screen we subtracted 160-bp [34], whereas for the 3% polyacrylamide screen we subtracted 10% of the target region at each end. This value was determined empirically as mutations were only detected in the central 80% of the target sequence (effective target region) for all genes (Figure 3).

**Table 4:** Comparison of the mutation frequencies obtained through the LI-COR and polyacrylamide/ethidium bromide screening method.

		**LI-COR**			**Polyacrylamide/Ethidium bromide**		
			
**Gene**	**Region**	**Sequence screened (kb)**	**Mut**.	**Mutation Frequency**	**Sequence screened (kb)**	**Mut**.	**Mutation Frequency**
*WKS1*	Kinase	930.0	18	1/52 kb	842.3	20	1/42 kb
	START	852.5	28	1/30 kb	390.1^a^	15	1/26 kb
*WKS2*	Kinase	998.4	25	1/40 kb	897.0	25	1/36 kb
	START	1053.7	25	1/42 kb	941.3	14	1/67 kb

Total/mean		3835	96	1/39.9 kb	3071	74	1/41.5 kb

^a ^384 M_2 _plants were screened

Four target regions where examined in the same 768 M_2 _plants from the hexaploid TILLING population and the total sequence screened was adjusted according to each method (see text).

Using the LI-COR detection technology we estimated a mutation frequency of 1 mutation per 40 kb (96 mutants in 3.84 Mb screened), whereas using the polyacrylamide setup we estimated 1 mutation per 41.5 kb (74 mutants in 3.07 Mb screened). Overall, each method detected two or three mutants not detected by the other method, but the majority of the mutations were detected by both. The exception to this was the *WKS2 *START domain target region in which the LI-COR screen identified eleven additional mutations (Table 4). Despite this, the average mutation frequencies for the four targets were almost identical. This suggests that when using fourfold pools (eight-fold dilution for mutations in heterozygous individuals), the polyacrylamide/ethidium bromide set-up has similar sensitivity to detect SNPs compared to the LI-COR platform, although there may be slight differences depending on the target region.

## Discussion

### Characterization of the EMS mutagenized populations

The use of reverse genetic approaches to determine gene function will become increasingly important as large amounts of sequence information become available in wheat. In an effort to address this, we created EMS-induced TILLING libraries in tetraploid and hexaploid wheat. We use the tetraploid TILLING population to generate mutants for basic research projects because it is easier and faster to generate complete null mutants. A single generation of crosses between A and B genome mutations, followed by selection of homozygous double mutants in the F_2 _populations is sufficient to generate null mutants. However, when a targeted mutant has important commercial applications (e.g. the *sbeIIa *mutants with predicted high amylose phenotype [35]) we screen the hexaploid TILLING population for mutations, because hexaploid wheat represents most of the wheat grown around the world (~95%) [36].

Characterization of these populations through the screening of several targets revealed mutation densities of at least one mutation per 49.4-kb and 68-kb in the hexaploid and tetraploid libraries, respectively. These mutation densities are lower than those found by Slade *et al*. [18] in wheat using similar EMS concentrations (one mutation per 24-kb and 40-kb screened in hexaploid and tetraploid libraries, respectively). The difference observed in these two studies is likely dependent on the different GC content of the target regions employed in the two studies, because EMS mutagenesis acts predominantly on GC residues [34]. Slade *et al*. [18] characterized their libraries by screening for mutants in the *waxy *genes. The regions included in their work have an unusually high GC content [*Wx-A1 *(56.4%), *Wx-B1 *(59.6%), *Wx-D1 *(55.4%)] whereas regions targeted in our study have an average GC content of 37.3% and 38.8% in the tetraploid and hexaploid targets, respectively. These values must be taken into account when estimating mutation densities because they represent the maximum number of mutations that can be found in those particular targets. For example, adjusting our reported mutation densities to the average GC content in Slade *et al*. [18] (55.9% hexaploid, 58.0% tetraploid) would yield new densities of one mutation per 34-kb and 44-kb for the hexaploid and tetraploid libraries, respectively. For future studies involving species where EMS mutagenesis is limited to GC>AT changes, it would be beneficial to report mutation densities corrected for a 50% GC content, or specify the GC content of the target regions, to allow for more meaningful comparisons. Applying this criterion, our mutation densities would be one mutation per 38-kb and 51-kb for the hexaploid and tetraploid libraries, respectively, in a target with 50% GC content.

Independent of the GC content, our reported mutation densities were lower than those reported by Slade *et al*. [18], by 43% in the hexaploid and 9% in the tetraploid population. The response of different hexaploid genetic backgrounds to EMS could account for some differences between the hexaploid libraries. This explanation cannot be applied to the tetraploid libraries since both studies used the same cultivar Kronos. Slight differences in EMS concentration and treatment conditions, environmental effects and experimental differences in the detection methods for both studies (for both LI-COR and 3% non-denaturing polyacrylamide) could account for the remaining variation.

Wheat is especially well suited for TILLING because of the tolerance of recently evolved polyploid species to high mutation densities [36]. The vast majority of greenhouse grown plants was fertile and displayed no apparent mutant phenotype. The mutation frequencies for wheat reported here and by Slade *et al*. [18] are five to ten times higher than mutation rates found in diploids such as barley, pea and Arabidopsis [25,37,38]. This high mutation frequency facilitates the identification of large allelic series in target genes using relatively small TILLING populations. For example, by screening 1,536 lines for a 1625-bp target region (1300-bp effectively screened, 50% GC content) we would expect to recover approximately 52 mutant alleles in the hexaploid library and 39 mutant alleles in the tetraploid library. Analysis of the mutations obtained in this study confirmed that the frequencies predicted by CODDLE were accurate and can be used to estimate the expected proportion of the different types of mutations to be recovered.

In an average TILLING fragment, truncation mutations are expected in 4 to 5% of the cases. Therefore, the large number of mutant alleles expected when TILLING an effective 1.3 kb region (1625-bp total target, 50% GC content) provides a high probability (>90% in hexaploid and 84% in tetraploid; *P *= [1-(1-0.045)^number of alleles^]) of obtaining at least one truncation mutation. In our screen using targets with lower GC content (<40%) we found truncations for 71.4% and 75% of the targets in the hexaploid and tetraploid libraries, respectively. This probability will vary according to GC content and can be improved by increasing the size of the target region if the gene is large enough (for example TILLING two regions of the same gene).

### Strategies for TILLING in polyploid genomes

The high probability of identifying truncation mutants is very important in a polyploid species, such as wheat, where the phenotype of a single mutant may be masked by the wild-type homoeologue present in another genome. Because of gene redundancy, it is generally necessary to cross single mutants in the A and B genome homoeologues to obtain a functional knockout in tetraploid wheat or create the triple A/B/D mutant in hexaploid wheat. Employing missense mutations in these lengthy genetic schemes is risky because if one of the mutations is not effective, it may be sufficient to limit the effect of the combined mutations on function. For the A and B genomes, the search for nonsense or splice junction mutations can involve both tetraploid and hexaploid TILLING populations, since mutations can be transferred by crossing. Hybridization of Kronos and UC1041 produces a pentaploid F_1_, which can be used as a female in subsequent backcrossing until fertility is restored.

Bioinformatics algorithms such as SIFT [39] and PSSM can be used to prioritize mutants for phenotypic evaluation, as reported before for *WKS1 *[32]. All five mutations with significant PSSM and SIFT scores were loss-of-function mutants of the resistance gene that led to susceptibility to the causal agent of stripe rust, *Puccinia striiformis *f. sp. *tritici *(Table 2). The only mutant line that remained resistant was T6-89 that had a non-significant SIFT score (0.46) and a borderline significant PSSM score (10.4). Despite this successful example for the use of SIFT and PSSM, the decision of using a missense mutation should be weighed against the amount of time and work that would be invested in producing double and triple mutants. The optimum strategy will depend on the objective and the gene being studied, as in some cases homoeologues are naturally deleted (as was the case for *WKS1*) or are not expressed.

The high mutation density in our libraries also implies that any given individual is predicted to carry between 260,000 (tetraploid) and 415,000 (hexaploid) mutations. Since most of the wheat genome (>83%) is represented by highly repetitive elements, and likely less than 3% of the wheat DNA encodes for genes (assuming a similar gene space per genome as Arabidopsis), most of the mutations will be outside the genes. Even after correcting for repetitive regions, for coding sequence space within a gene, and for the proportion of mutations that result in missense or truncations, each individual from the TILLING population is expected to carry thousands of missense mutations and hundreds of truncations. A simple way to reduce this large amount of background mutations is to backcross the mutants to the non-mutagenized recurrent parent for two to three generations. These backcross generations are essential when the mutations are being used for wheat breeding because the background mutations can reduce the average performance of the populations generated directly from crosses with the original mutants. The mutant SNP can be tracked in the backcrossing scheme by direct sequencing of the genome-specific amplicon in each generation. Alternatively, many SNPs lead to polymorphisms in restriction sites which can be used to develop Cleavage Amplified Polymorphism (CAPs) markers. Alternatively, derived CAP (dCAP) markers can also be designed [40]. Ultimately, the most effective strategy will depend on the costs of sequencing and restriction enzymes for each lab.

For research projects with a clear phenotype, selecting for sister lines homozygous for the presence and absence of the mutation is an effective strategy. Sister lines with and without the mutations share many of the same background mutations, thus serving as a better control than the wild-type line with no background mutations. This approach is especially powerful when multiple sets of independent sister lines are examined [32] as the probability of finding mutations by chance in a linked gene is extremely low. For example, with the mutation densities of the hexaploid population (1 mutation per 38 kb), the probability of finding at least one an amino acid change in any 1.5 kb coding region is 2.5% ({1- [(1-(1/38000)]^1500^}*0.66); as 66% of GC>AT codon changes are non-synonymous]. If two or three independent lines were examined, then this probability drops dramatically (*P *< 0.0007 and *P *< 0.00002, respectively).

### Primer design in polyploid species

Primer design is an important aspect of TILLING in polyploid species. Genome specificity needs to be combined with a high yielding PCR product for proper mutant detection. The first step in designing genome specific primers is the sequencing of the different homoeologous copies. These sequences can be obtained for highly expressed genes by a bioinformatics characterization of available wheat ESTs. Alternatively, genome sequences can be generated by screening the BAC libraries and sequencing from individual BACs or by sequencing the diploid donors of the different wheat genomes. We routinely use *T. urartu *for the A genome, *Ae. tauschii *for the D genome (accession AL8/78 closely related to the D genome of wheat) and *T. speltoides *as the best approximation to the B genome. If better sequences for the B genome are required, a fast strategy is to clone and sequence several clones from PCR products obtained from tetraploid wheat.

The optimal target regions were defined by the CODDLE program using the following criteria: a) mutations close to primers (~10% of target sequence) are not readily detected, particularly in large amplification products b) maximize exons and/or intron-exon splice junctions and c) maximize regions encoding for conserved domains within the protein. Primers are usually designed in the introns or 5' or 3' UTRs flanking the target exons as these regions are more polymorphic (important for genome specificity).

**Figure 4:**
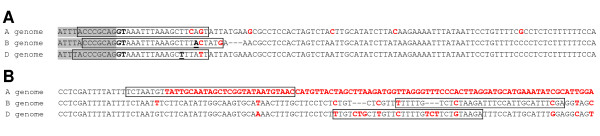
**Alignment of homoeologous *SBEIIa *sequences used to design genome-specific forward (A) and reverse (B) primers**. Primers are surrounded by boxes and genome specific polymorphisms are indicated in bold red. Exon 4 is in grey highlight and all other sequence corresponds to intron 4 (**A**) or intron 9 (**B**). Bold underlined bases in panel **A **indicate positions of introduced mismatches in primers relative to the genomic sequence. In/del events are represented by dashed lines except in the A genome of intron 9 (**B**) which has a large in/del event relative to the B and D genomes that is represented by bold red letters.

Different strategies can be used to generate genome-specific primers (Figure 4) [41,42]. If one of the primers can overlap a unique in/del or multiple intergenomic SNPs, this is usually sufficient to generate genome specificity (e.g. *SBEIIa *A and D genomes, *SBEIIb *B genome). In other cases, where there is lower polymorphism between homoeologues, both primers can be designed such that the first nucleotide from the 3' end of the primers aligns to genome-specific SNPs (e.g. *SBEIIb *A genome). In these cases, increased specificity can be attained by introducing a mismatch in the primer at the third or fourth position from the 3' end. Although this generates a mismatch between the target sequence and the primer (at the third or fourth position from the 3' end), the two mismatches with the other homoeologues increase the probability of genome-specific amplification. These strategies can be combined as in the *SBEIIa *B genome primers (3' end SNP, unique in/del overlap, and introduced mismatch; Figure 4) and used in conjunction with touchdown PCR to increase specificity.

### Non-denaturing Polyacrylamide Detection Method

We report here the use of a modified screening technique that can be used to detect mutations in TILLING populations. We found equivalent mutations using the LI-COR and 3% non-denaturing polyacrylamide, suggesting that this system represents a viable low-cost alternative to the current technologies. Our 3% non-denaturing polyacrylamide system is based on ethidium bromide staining, eliminating the need for the genotyping instrument and fluorescently labeled primers. This is especially relevant in polyploid genomes as the fluorescent label attached to primers can reduce their genome specificity, requiring additional PCR optimization. Samples can be loaded directly after stopping the CJE digestion reaction with 0.225 M EDTA, eliminating the subsequent steps of sample purification and volume reduction required in LI-COR screens. We also found that by extending and optimizing the CJE digestion time (determined empirically, results not shown) we observed digestion of both strands, despite *Cel*I being a single strand mismatch-specific endonuclease. This additional CJE activity eliminates the need of denaturing polyacrylamide gels which are more time consuming and technically more difficult than a non-denaturing system. Although we are able to increase the size of our target regions to over 1.5-kb, we were unable to find mutations in the first 10% of the sequences adjacent to each primer. Therefore the total sequence screened is roughly similar to the LI-COR method (1.3 to 1.4-kb) with the disadvantage that more sequence information is needed in our method to accommodate the larger distance between the primers and the region where mutations are effectively detected.

An additional advantage of the LI-COR system, is that the use of different dyes for each primer allows a precise estimation for the location of the mutant. In our set-up, two possible locations are estimated since we have no information as to whether the estimated distance is from the forward or the reverse primer. This implies an additional cost for sequencing a larger number of mutants to identify EMS-induced polymorphism in the desired regions. Another possible drawback of this set-up is the need for manual analysis of the gels since no software has been developed for this system. Despite this, gel image analysis requires approximately 20–30 minutes, similar to the time required with GelBuddy or similar gel analysis programs.

For both libraries we found a number of duplicate mutations, as well as a few triple and quadruple mutations, that were higher than expected by chance. These mutations are likely residual polymorphisms in the mutagenized seed, originated from residual heterozygous alleles in some of the plants used for the production of breeder's seed of Kronos or the seed stock for UC1041+*Gpc-B1*/*Yr36*. For example, we confirmed that the original seed stock of UC1041+*Gpc-B1*/*Yr36 *is polymorphic for a known 1-bp deletion in the coding region of the *VRN-D3 *allele [43]. These observations also suggest that the polyacrylamide detection method should be amenable for EcoTILLING [44].

The polyacrylamide detection method is especially relevant for species, such as wheat, that have no central TILLING service available. Even if a central service for wheat becomes available, individual researchers may need to TILL genotypes carrying specific alleles (such as for disease resistance) that may be absent in available TILLING populations. Although several alternative detection methods have been published, most rely on expensive equipment (sequencers, HPLC, gene analyzers) that precludes many individual laboratories from performing TILLING. The development of a non-denaturing polyacrylamide detection system makes TILLING more accessible to a larger set of researchers and breeding programs and may facilitate the development of multiple wheat TILLING populations. We plan to make the DNAs of our TILLING lines available on a cost recovery basis for other research groups to screen [45]. This should enable different research groups to screen for mutations in their gene of interest and expand their capabilities for wheat functional genomics.

We have pursued further characterization of over 20 mutants for the *WKS *[32] and *SBEII *genes. Although the phenotypic characterization of the *SBEII *mutants is beyond the scope of this work, we have successfully confirmed each *WKS *and *SBEII *mutation in its corresponding M_3 _seed stock. This is an important final validation step, suggesting that the two libraries will be an effective reverse genetics resource.

Other groups are also currently developing TILLING resources in wheat. These include tetraploid libraries in variety 'Cham 1'[46] as well as hexaploid libraries in several varieties ('Alpowa', 'Louise' and 'Jagger' in the USA (Camille M. Steber, personal communication), Cadenza [46] and 'QAL2000' and 'Ventura' in Australia [21]). The generation of multiple TILLING libraries in wheat will allow an even greater flexibility and robustness to wheat TILLING as any targets which are missing in one population could be screened for in others.

## Conclusion

We show here that TILLING constitutes an effective way to screen large wheat mutant libraries for induced polymorphisms. The high mutation densities tolerated by recently evolved polyploids makes this approach especially attractive as a reverse genetics platform for wheat. The development of a low-cost detection method that has similar assay sensitivity to the LI-COR technology, together with public access to these TILLING populations, will likely make this technology more accessible. We hope that researchers will use these libraries to create novel allelic diversity for breeding pasta and common wheat and to better understand basic gene function in these important crop species. We think that the new TILLING resources will shift the paradigm of what can be done in functional gene analysis in wheat.

## Methods

### Mutagenesis and population development and growth conditions

For the EMS treatments, 10–20 g of seeds were placed in 1 L plastic buckets with 40 mL 10% Tween 20 solution. The buckets were placed on an orbital shaker for 15 minutes at 112.5 rpm after which the solution was discarded. 250 mL of tap water were added to each bucket to wash off excess Tween 20 and they were then placed on the orbital shaker at 112.5 rpm for 5 minutes. The water was discarded and this washing step was repeated three more time. A final volume of 250 mL of tap water was used for the EMS treatment and the buckets were placed on the orbital shaker at 112.5 rpm for 18 hours at room temperature. After EMS treatment, seeds were thoroughly washed with tap water for 3 hours and then placed at 4°C for 5 days before being transferred to room temperature.

The EMS-treated seeds (M_1_) were sown in individual cones with soil and the M_2 _seeds from each individual plant were collected and labeled. Designators 'T6' and 'T4' were used for the hexaploid and tetraploid lines, respectively. Five M_2 _seeds per line were sown in one pot and they were later thinned to leave a single M_2 _plants per line.

### DNA isolation and preparation of plates for pooling

Genomic DNA was isolated from 1,386 and 1,536 M_2 _plants for the tetraploid and hexaploid populations, respectively, using a previously published protocol [47]. This large-scale DNA extraction protocol yields approximately 1–2 mg of DNA and was selected to generate abundant DNA of excellent quality for distribution. DNA concentrations were measured using a spectrophotometer (Nanodrop, Thermo Scientific, Wilmington, DE, USA) and standardized. Using equivalent amounts of DNA from individual plants, samples were pooled fourfold and organized into 96-well format. A total of four 96-well plates were built for each TILLING population, representing the 1,386 and 1,536 M_2 _plants.

### Primer design

#### *WKS1 *and *WKS2*

Primers were designed based on the sequenced BAC contig including *WKS1 *and *WKS2 *[Genbank: EU835198]. Only the hexaploid population was screened for mutations as these genes are absent in the tetraploid line. Two regions were screened for each gene. The first one included the complete kinase domain and was 1,371-bp and 1,460-bp in *WKS1 *and *WKS2*, respectively. The second region included part of the START domain and was 1,270-bp and 1,532-bp in *WKS1 *and *WKS2*, respectively.

#### SBEIIa

This gene was screened in both the tetraploid and hexaploid populations. The *Aegilops tauschii *sequence for *SBEIIa *was previously deposited in Genbank [AF338431] and served as the D genome template for this gene. The A and B genome sequences were obtained by screening a tetraploid BAC library [8] with a combination of probes for different SBE genes. The addresses for positive clones were kindly provided by Ravi Chibbar (University of Saskatchewan, Canada) and Yong Qiang Gu (USDA-ARS). Based on the *Ae. tauschii *sequence, we designed primers (SBEIIa_F1: TCGTGCTGCTATTGACCAAC, SBEIIa_R1: TGGAGTTCCAAAACGGCTAC) to amplify the region surrounding the glycogen branching enzyme domain (cd02854–5) (exons 5–9) in the SBE positive clones. These primers amplified 6 clones which were classified into two types according to their amplification size. Type I clones (168P02, 696H16, 788M03, 1287F23) amplified a fragment of less than 2,500-bp [GenBank: GQ254775], whereas type II clones (284D01, 1133N09) amplified a fragment larger than 2,500-bp [GenBank: GQ254772]. Clones from each group were sequenced and primers specific to each type were designed (see Additional file 1). Using the nulli-tetrasomic lines (N2AT2B, N2BT2D, N2DT2A) we assigned type I clones to the B genome and type II clones to the A genome. The *SBEIIa *sequences of Kronos and UC1041+*GpcB1/Yr36 *are deposited in GenBank [GQ254773–GQ254774, GQ254776–GQ254777]

#### SBEIIb

This gene was screened only in the tetraploid population. The *Aegilops tauschii *sequence for *SBEIIb *was previously deposited in Genbank [AY740398] and served as the D genome template for this gene. Based on the *Ae. tauschii *sequence we designed primers (SBEIIb_F1: TGAAGACACGAGCAGAATGG, SBEIIb_R1: CCAAGTCTTTTAATTCTTGGAAGC) to amplify the region surrounding the glycogen branching enzyme domain (cd02854–5) (exons 5–9) in the SBE positive clones. These primers amplified 10 clones which were classified into two types according to their amplification size. Type I clones (151G21, 187B09, 239D20, 416A19, 977N16, 1207H21) amplified a larger fragment [GenBank: GQ254779] compared to type II clones (603K14, 731E19, 1042I07, 1005M11) [GenBank: GQ254778]. Clones from each group were sequenced and primers specific to each type were designed (see Additional file 1). Using the nulli-tetrasomic lines (N2AT2B, N2BT2D, N2DT2A) we assigned type I clones to the B genome and type II clones to the A genome. *SBEIIb *sequences of Kronos are deposited in GenBank [GQ254780–GQ254781]

### Screening technique and two-step strategy

The protocol for the LI-COR detection method has been previously published [48]. The protocol for the targets visualized through the polyacrylamide detection methods includes a two-step screening approach. The first PCR screen of the complete set of DNA pools was carried out in a 25-μl reaction volume using 50–100 ng of pooled DNA (a large amount of DNA is required given the large genome size of wheat), 1 U of Taq polymerase and the following cycling conditions: initial denaturation at 95°C for 2 min, followed by 14 cycles of touchdown at 94°C for 20 s, from 64 to 57 for 30 s (0.5°C decrease per cycle) and extension at 72°C for 75 s. This touchdown cycle is followed by 37 cycles of 94°C for 20 s, 57°C for 30 s and 72°C for 75 s. A denaturing and re-annealing step is included at the end of the PCR reaction (99°C for 10 min, 70 cycles of 70°C for 20 s decreasing 0.3°C per cycle) to allow the formation of heteroduplexes if a mutation is present in the pool.

After PCR amplification, 12 μl of sample (~500 ng) was digested with celery juice extract (CJE) which was obtained using the protocol described by Till *et al*. [48]. Due to the inherent variability of different celery juice extracts, the optimal amount of CJE for heteroduplex-digestion was determined empirically [48] using targets with known mutations. This was done by performing TILLING reactions at 45°C for 30 minutes, but with varying amounts of CJE. Using too little CJE results in a strong top band (corresponding to the PCR reaction), but no visible lower bands (corresponding to the cut fragments). Too much CJE leads to complete digestion of the original PCR product resulting in a smear along the gel. A good signal-to-noise ratio is depicted in Figure 1A and 1C. Digestions for this study included: 12 μl PCR product, 0.12 μl CJE, 1.7 μl 10× digestion buffer [48] and 3.18 μl dH_2_O for a final volume of 17 μl. The digestion was carried out at 45°C for 30 minutes and stopped immediately by adding 5 μl of 0.225 M EDTA per sample and mixing thoroughly. Two micro liters of bromophenol blue loading dye were added and the complete volume (24 μl) was loaded on the gel.

Samples were visualized on a 3% polyacrylamide gel (19:1 Acrylamide:bis ratio) in 0.5× TBE running buffer with ethidium bromide. We use a 100-lane vertical electrophoresis system (gel size: 22 cm tall by 1.5 mm thick; CBS Scientific, Del Mar, CA, USA) which was run at 350 V for 45–60 minutes. Gel images were analyzed manually on PowerPoint (Microsoft Corp., Seattle, WA, USA) and positive pools were identified by the presence of cleaved products whose combined size was similar to the original PCR product.

The second screen was performed using individuals DNAs only from the DNA pools that showed cleaved products in the first screen. PCR amplifications were as described before with the exception that the heteroduplex formation step was postponed. First, 6 μl from each PCR sample were pooled in four pairs (*a+b*, *c+d*, *a+c *and *b+d*) following the diagram in Figure 1B. The heteroduplex formation step was then performed on the mixed pairs and the samples followed the same detection protocol as described above. The pooling step is necessary to detect homozygous mutations in the M_2 _plants since combining two samples allows heteroduplex formation and detection which would otherwise go undetected in a single homozygous sample. In addition to the identification of the individual from the pool that carries the mutation, this step also provides an independent validation of the mutations and a better estimation of its location within the target region.

### Sequence analysis

To characterize the individual mutations, the residual PCR product from the selected individuals was used as a template for sequencing. PCR products (2 μl/sample) were cleaned using ExoSAP-IT (USB Corp., Cleveland, OH, USA) according to manufacturer's instructions, and subsequently sequenced using BigDye Terminator sequencing kit and an ABI -3730 DNA Sequencer (Applied Biosystems, Foster City, CA, USA).

## Abbreviations

BAC: bacterial artificial chromosome; CAP: cleavage amplified polymorphism; CJE: celery juice extract; CODDLE: choose codons to optimize the detection of deleterious lesions; dCAP: derived cleavage amplified polymorphism; EMS: ethyl methane sulphonate; EST: expressed sequence tags; PCR: polymerase chain reaction; PSSM: position-specific scoring matrix; PST: *Puccinia striiformis *f. sp. *tritici*; *SBE*: *Starch Branching Enzyme*; SIFT: sorting intolerant from tolerant; TILLING: targeted induced local lesions in genomes; *WKS*:*Wheat Kinase Start*.

## Authors' contributions

CU, LC and JD planned and headed the development of the mutant populations. CU and FP were responsible for population development, DNA extraction, sample normalization and arraying, and seed stock organization. CU and FP isolated the *SBE *genomic sequences and designed and tested primers. CU and JD developed the non-denaturing polyacrylamide screening method. CU, FP and PC performed the non-denaturing polyacrylamide screens, interpreted the mutation detection data and performed sequencing reactions. RT, HT and SB performed the LI-COR screens. CU, LC and JD conceived the study, obtained funding for it and were primarily responsible for drafting and revising the manuscript with contributions from co-authors. All authors read and approved the final manuscript.

## Supplementary Material

Additional file 1 **Primers used for TILLING screen**. A table displaying the Primers used for TILLING screen. Click here for file
