# Supplementary material for: A modified TILLING approach to detect induced mutations in tetraploid and hexaploid wheat
Source: BMC Plant Biol. 2009 Aug 28;9:115. doi: 10.1186/1471-2229-9-115 (PMC2748083; doi:10.1186/1471-2229-9-115)
Supplement: Additional file 1 — Primers used for TILLING screen. A table displaying the Primers used for TILLING screen. [file 1471-2229-9-115-S1.pdf]

Additional File 1: Primers used for TILLING screen

Gene	Domain/Chr	Primer Sequence
WKS1	Kinase	AAGAATAAAATTGGTTTTTAATTTCGGAAAAGGTC<br>ATGGAGGTGTTGGCTTTTGTGAGATGTTT
	START	TGCTGGAACTTGGAGCCATATAAAAATGC<br>TGAACGGAGGGAGTGTTAACTAGCATAGG
WKS2	Kinase	GCCATGAACAACGAACAATCACACGATA<br>TAAGTTGTTACTCAGCCCCAGCGCAATAC
	START	TCTGCTCCCAGACCCACCTCATACTTAAA<br>GCAAAAGAGAAAAATGTTAAGCAGCGGAAA
SBEIIa (6x)	2A	ACCCGCAGGTAAATTTAAAGCTTCAGT<br>GTTACATTATACCGAGCTATTGCAATAACATTAGA
	2B	CCCGCAGGTAAATTTAAAGCTTT**G**CTATG[1]<br>TCGAAATGCAATGGAAATCTTAGAGACAAAAA
	2D	TACCCGCAGGTAAATTTAAAGC**G**TTATT[1]<br>TCTTACAGAAGACAAAAGAACAAGCAGACAA
SBEIIa (4x)	2A	ACCCGCAGGTAAATTTAAAGCTTCAGT<br>TGCATCCTAAGTGGGAAACC
	2B	CCCGCAGGTAAATTTAAAGCTTT**G**CTATG[1]<br>TCGAAATGCAATGGAAATCTTAGAGACAAAAA
SBEIIb (4x)	2A	TGCAATGTGAGCTACCGATAAG<br>GACTGAAATGACACTATTCCAGCA
	2B	TGGACGGGTGTTCTTTTACC<br>AGTTACATCAAGTTAAACATTTGACCA

[1] Bold underlined bases indicate positions of introduced mismatches with respect to the genomic sequence.
